# *Fas* mutation reduces obesity by increasing IL-4 and IL-10 expression and promoting white adipose tissue browning

**DOI:** 10.1038/s41598-020-68971-7

**Published:** 2020-07-20

**Authors:** Eun Wha Choi, Minjae Lee, Ji Woo Song, Kyeongdae Kim, Jungmin Lee, Jehoon Yang, Seo Hyun Lee, Il Yong Kim, Jae-Hoon Choi, Je Kyung Seong

**Affiliations:** 1 Department of Veterinary Clinical Pathology, College of Veterinary Medicine and Institute of Veterinary Science, Kangwon National University, 1 Kangwondaehak-gil, Chuncheon-si, Gangwon-do 24341 Republic of Korea; 2 Laboratory Animal Research Center, Samsung Biomedical Research Institute, Samsung Medical Center, 81 Irwon-ro, Gangnam-gu, Seoul, 06351 Republic of Korea; 3 Department of Life Science, College of Natural Sciences, Hanyang University, 222 Wangsimni-ro, Seongdong-gu, Seoul, 04763 Republic of Korea; 4 Laboratory of Developmental Biology and Genomics, BK21 Plus Program for Advanced Veterinary Science, Research Institute for Veterinary Science, College of Veterinary Medicine, and Korea Mouse Phenotyping Center, Seoul National University, 1 Gwanak-ro, Gwanak-gu, Seoul, 08826 Republic of Korea; 5 Interdiscplinary Program for Bioinformatics, Seoul National University, 1 Gwanak-ro, Gwanak-gu, Seoul, 08826 Republic of Korea

**Keywords:** Obesity, Experimental models of disease

## Abstract

Brown adipose tissue generates heat via the mitochondrial uncoupling protein UCP1 to protect against obesity and hypothermia. *Fas* mutant MRL/lpr mice exhibit a significantly leaner phenotype compared to wild type MRL/MpJ mice. In this study, we evaluated the inflammatory cell population in the adipose tissue of MRL/lpr mice, which could potentially influence their lean phenotype. Furthermore, we compared beige fat activity between the MRL/MpJ and MRL/lpr mice. *Fas* mutation resulted in high body temperature, improved glucose tolerance, and decreased fat mass and adipocyte size. *Fas* mutation prevented high-fat diet-induced obesity and decreased the white adipose tissue M1:M2 ratio. When mice were fed a high-fat diet, *UCP1*, *IL-4*, *IL-10*, and tyrosine hydroxylase genes had significantly higher expression in *Fas*-mutant mice than in wild type mice. After a cold challenge, UCP1 expression and browning were also significantly higher in the *Fas*-mutant mice. In summary, *Fas*-mutant mice are resistant to high-fat diet-induced obesity due to increased IL-4 and IL-10 levels and the promotion of thermogenic protein activity and browning in their adipose tissues. STAT6 activation might contribute to M2 polarisation by increasing IL-4 and IL-10 levels while increases in M2 and tyrosine hydroxylase levels promote browning in response to *Fas* mutation.

## Introduction

Weight gain and obesity are the main risk factors of type 2 diabetes mellitus, myocardial infarction, stroke, and atherosclerosis^1,2^. Excess adipose tissue accumulation is accompanied by local inflammation characterised by the infiltration of inflammatory cells^3^.

Brown adipose tissue (BAT) is a key site for heat production in mammals and it has been previously targeted to promote weight loss^4,5^. BAT generates heat via the action of the mitochondrial uncoupling protein 1 (UCP1) to protect against obesity and hypothermia^6^. Increasing the metabolic activity of beige fat, brown fat, or both is essential for the treatment of obesity and metabolic diseases^7^. Interestingly, increased activities of brown and beige adipocytes have been linked to obesity resistance in several genetically engineered mouse (GEM) models^7^.

The Fas receptor (Fas) is a member of the tumour necrosis factor (TNF)-receptor family. Fas/Fas ligand (FasL) interactions initiate apoptosis by inducing the activation of the Fas-associated death domain and the cleavage of caspase 8^8,9^. Apoptosis mediated by Fas-FasL is a key mechanism for immune homeostasis and Fas plays an essential role in preventing autoimmunity by the deletion of potentially pathogenic autoreactive lymphocytes from the blood and tissues to maintain lymphocyte homeostasis and peripheral immune tolerance^10^. According to a previous study, *Fas* expression was increased in adipocytes isolated from insulin-resistant mice, such as *db/db* and *ob/ob* mice, and in the adipose tissues of obese and diabetic patients^11^.

Mice that are homozygous for the lymphoproliferation spontaneous mutation (*Fas*^*lpr*^) exhibit systemic autoimmunity; the onset and severity of the clinical symptoms associated with this gene are strain-dependent. Additionally, MRL-Mp-*Fas*^*lpr*^/*Fas*^*lpr*^ mouse (MRL/lpr) have a higher proportion of spontaneous anti-dsDNA antibodies production, more severe glomerulonephritis, and shorter life span than other strains (C57BL/6J and C3H/He background). From approximately three months of age, the levels of circulating immune complexes in MRL/lpr mice rise significantly compared to those in the MRL normal mice (MRL/MpJ)^12^. MRL/lpr (*Fas* mutation) mice are one of the most important models of human systemic lupus erythematosus (SLE)^13–15^ and are characterised by splenomegaly and lymphadenopathy due to severe infiltration by autoreactive lymphocytes.

SLE is an autoimmune disease that causes the immune system to produce antibodies against self-antigens, particularly the proteins within the cell nucleus; these antibodies attack healthy tissues in many parts of the body. Common symptoms of SLE include fever, fatigue, glomerulonephritis, arthritis, swollen lymph nodes, hair loss, and dermatitis (a red rash on the face). Although the cause of SLE is unknown, the involvement of genetic factors acting in concert with hormonal and environmental factors is speculated^16^.

When adipose tissue was obtained from SLE mice to generate adipose tissue-derived mesenchymal stem cells, the amount of adipose tissue obtained from MRL/lpr mice was significantly less than that obtained from MRL/MpJ mice.

GEM refers to mice in which a specific gene has been removed or modified using genetic engineering techniques. GEM models could be characterised as transgenic (Tg) or knockout (KO) mice. In Tg mice, DNA is randomly inserted into the genome by pronuclear injection into a single cell of the mouse embryo^17^, whereas the generation of KO mice involves inactivation of an existing gene by replacing or disrupting it with a synthetic DNA sequence^18^. Tg and KO mice are used extensively in research as models of human diseases and are also used for drug development, as they facilitate target validation^19^.

In many GEM KO mice, although lean phenotypes are not evident when they are fed a chow diet, they tend to resist obesity during HFD feeding. However, in MRL/ MpJ mice, a lean phenotype was evident in mice possessing a *Fas* mutation when they were fed a chow diet and housed at room temperature.

Wheest et al. reported that the deletion of *Fas* in adipocytes decreased adipose tissue inflammation, hepatic steatosis, and insulin resistance induced by a high-fat diet (HFD). Compared to HFD-fed wild-type mice, the mRNA expression levels of IL-6, Monocyte Chemoattractant Protein-1 (MCP-1), CD11b, and resistin in white adipose tissue decreased remarkably while that of IL-10 increased markedly in HFD-fed adipocyte-specific *Fas*-KO (AFasKO) C57BL/6 mice^11^. Despite this observation, a comparative study between *Fas*-KO and wild type (WT) mice to examine the inflammatory cell populations has not been conducted.

The purpose of this study was to evaluate the population of inflammatory cells involved in the lean phenotype of MRL/lpr mice. The activity of beige fat was also compared between MRL/MpJ and MRL/lpr mice.

## Results

### *Fas* mutation mice were resistant to high-fat diet-induced obesity and exhibited enhanced thermogenesis compared to WT mice.

**Figure 1:**
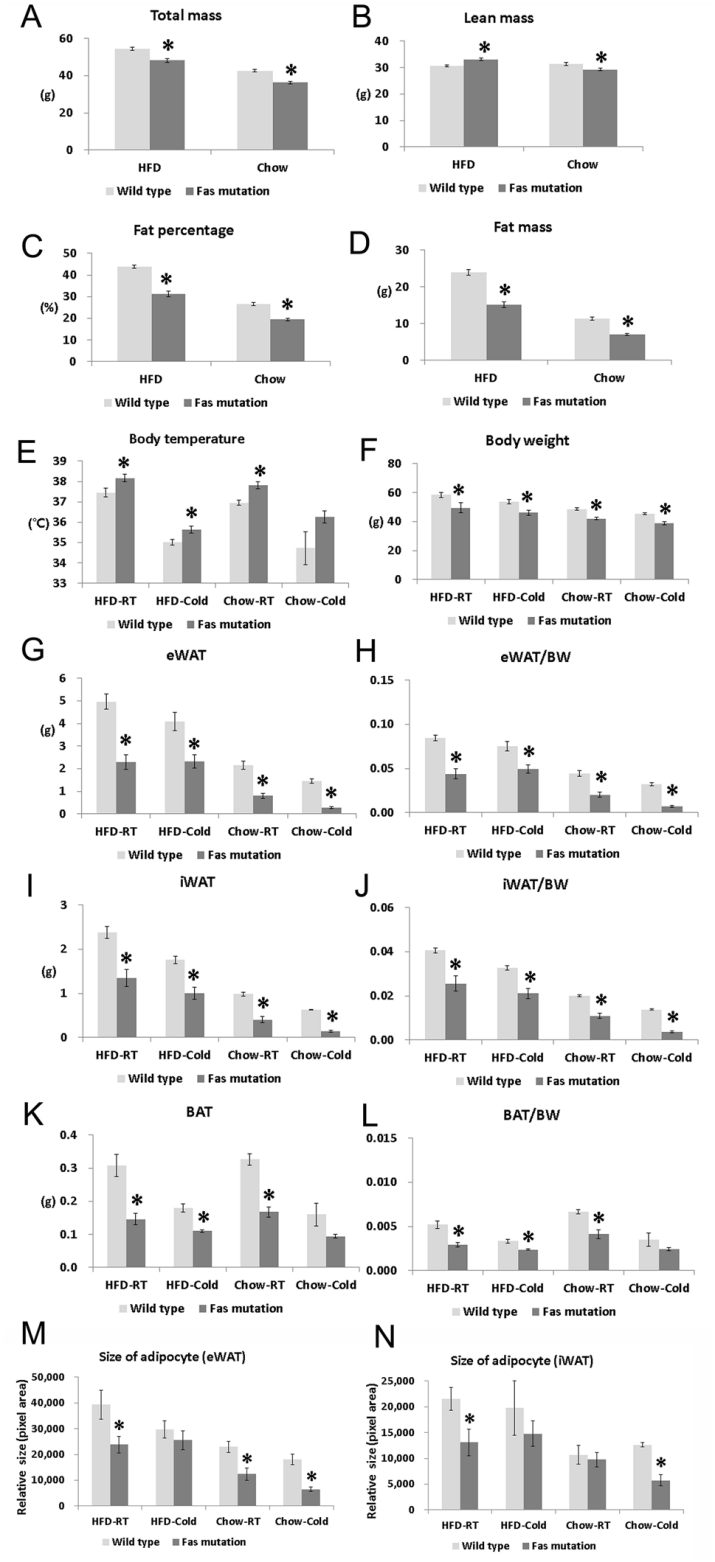
MRL/lpr (*Fas* mutation) mice exhibited a significantly lean phenotype compared to that of MRL/MpJ (wild type) mice. *Fas*-mutant mice were resistant to high-fat diet-induced obesity and exhibited enhanced thermogenesis compared to that of wild type mice. (**A**) Total mass (g) measured by dual-energy X-ray absorptiometry (DEXA), (**B**) lean mass (g) measured by DEXA, (**C**) the percentage of fat measured by DEXA, (**D**) total weight of fat measured by DEXA, (**E**) body temperature measured by monitoring system immediately prior to autopsy, (**F**) total body weight, (**G**) weight of epididymal white adipose tissue (eWAT), (**H**) eWAT:body weight (BW) ratio, (**I**) weight of inguinal white adipose tissue (iWAT), (**J**) iWAT:body weight ratio, (**K**) weight of brown adipose tissue (BAT), (**L**) BAT:BW ratio. (**M**) Relative sizes of adipocytes (pixel area) in eWAT were measured with Image J (Adiposoft). (**N**) Relative sizes of adipocytes in iWAT were measured with Image J (n = 5–8 per group). Data are represented as mean ± standard error of the mean (SEM). The student t-test was used to compare the means from two groups (Wild type vs. *Fas* mutation under the same food and environmental temperature condition). *indicates significant differences (p < 0.05) compared to wild type mice under the same conditions (diet and environment temperature).

The total mass, the weight of body fat, and the percentage of body fat measured by DEXA were significantly lower in the *Fas* mutation group than in the WT group (Fig. 1A–D). The body temperatures of *Fas* mutant mice were significantly higher than those of WT mice when the mice were fed with HFD and housed at 22 °C (HFD-RT) or 4 °C (HFD-cold) and when fed with chow diet and housed at 22 °C (chow-RT) (Fig. 1E).

Regardless of environmental temperature (22 °C or 4 °C) and type of diet (HFD or chow diet), the total body weight, weight of epididymal white adipose tissue (eWAT), inguinal white adipose tissue (iWAT), eWAT: body weight (BW) ratio, and iWAT: the BW ratio was significantly lower in the *Fas* mutation group than in the WT group (Fig. 1F–J). The weight of BAT and the ratio of BAT: the BW was also significantly lower in the *Fas* mutation group than in the WT group, except in the chow-cold condition (Fig. 1K,L).

The relative sizes of adipocyte in eWAT and iWAT after 11–12 weeks of HFD feeding were significantly smaller in the *Fas* mutation group than in the WT mice (Fig. 1M,N). Relative sizes of adipocyte in eWAT and iWAT after 48 h of cold challenge were also significantly smaller in the *Fas* mutation group than in the WT mice (Fig. 1M,N).

### *Fas* mutation mice exhibited enhanced energy expenditure

**Figure 2:**
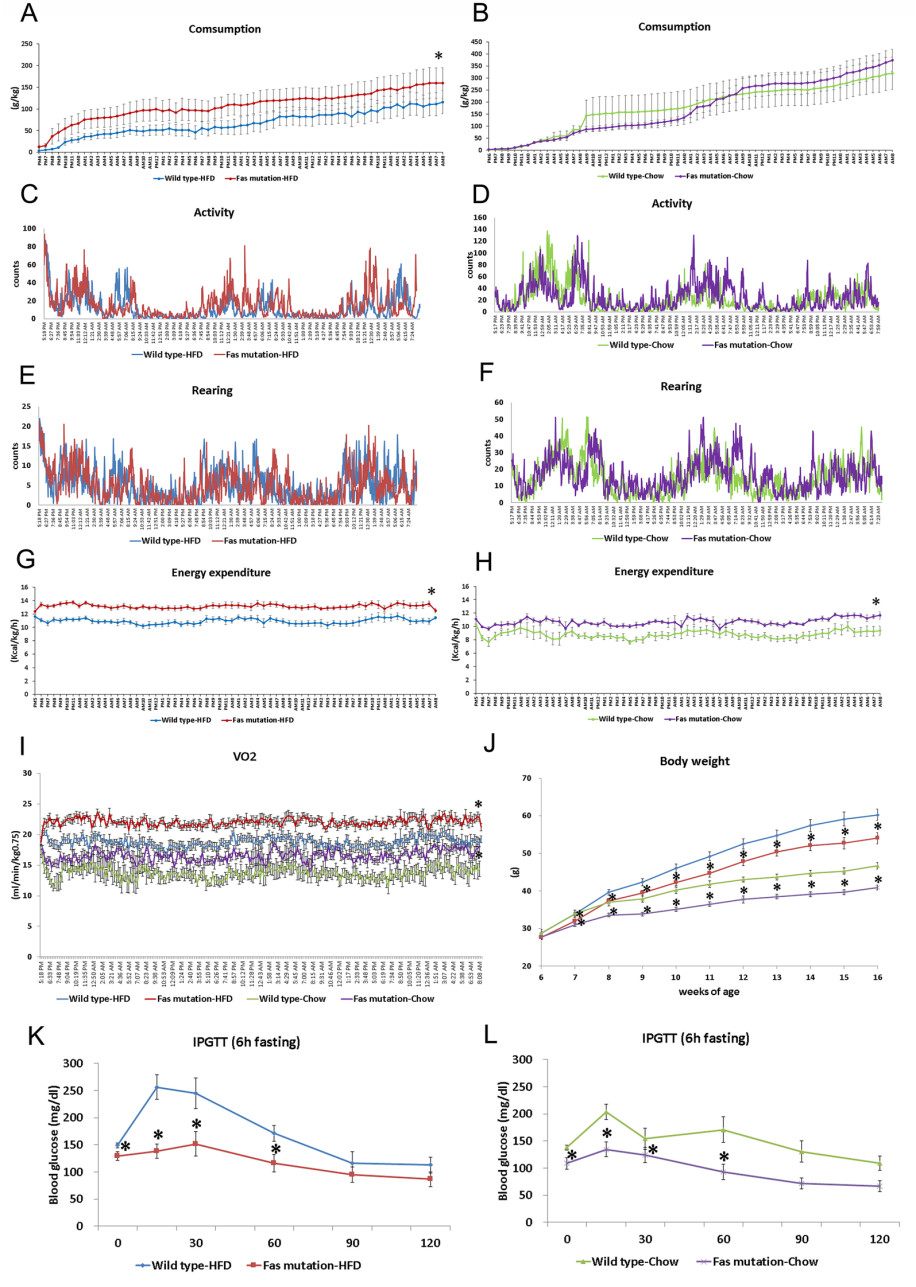
*Fas* mutation enhanced energy expenditure and glucose tolerance (**A**) Food intake normalised to body weight for the *Fas* mutation and control groups when mice were fed with high-fat diet (HFD); (**B**) food intake normalised to body weight when mice were fed with Chow diet; (**C**) activity when mice were fed with HFD; (**D**) activity when mice were fed with Chow diet; (**E**) rearing when mice were fed with HFD; (**F**) rearing when mice were fed with Chow diet; (**G**) energy expenditure when mice were fed with HFD; (H) energy expenditure when mice were fed with Chow diet; (**I**) oxygen consumption in response to different diets and for genotypes; (**J**) changes in body weight during the experiments; (**K**) intraperitoneal glucose tolerance test (IPGTT) results when mice were fed with HFD; (**L**) IPGTT results when mice were fed with chow diet (n = 8 per group). Data are represented as mean ± standard error of the mean (SEM). The data from calorimetry assays (**A**–**I**) were analysed by repeated measured one-way analysis of variance. The data from body weight and IPGTT assays (**J**, **K**, **L**) were analysed using the student t-test at each time point (Wild type vs. *Fas* mutation under the same food conditions). *Significant differences (p < 0.05) compared to wild type mice under the same conditions (diet).

Food intake normalized to body weight was higher in the *Fas* mutation group than in the control group when mice were fed with HFD (Fig. 2A,B). Activity and rearing were not different between the control and *Fas* mutation groups (Fig. 2C–F). Energy expenditure was significantly higher in the *Fas* mutation group than in the control group when mice were fed with HFD or chow diet (Fig. 2G–I). Changes in body weight during the experiment are presented in Fig. 2J. *Fas* mutation mice were significantly lighter in weight than WT mice at 7–16 weeks of age in the same food condition.

### *Fas* mutation enhanced glucose tolerance

After a 6 h fasting period, blood glucose levels were significantly lower in the *Fas* mutation group than in the control group (Fig. 2K,L). The time for the return to the baseline level for blood glucose was also shorter in the *Fas* mutation group than in the control group (Fig. 2K,L).

### *Fas* mutation promoted thermogenic protein expression in white adipose tissues

**Figure 3:**
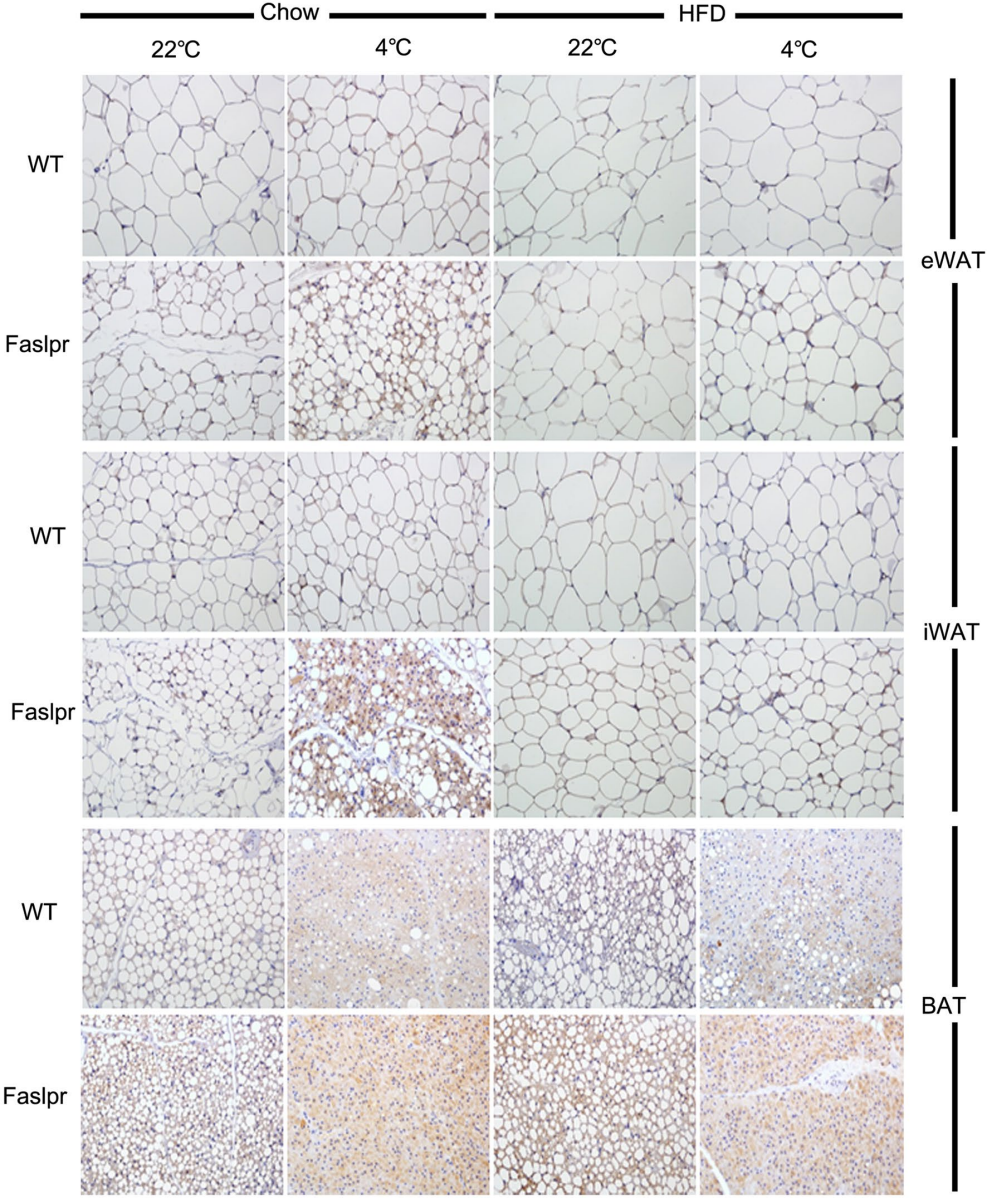
*Fas* mutation promoted thermogenic protein expression in white adipose tissues. Representative adipose tissue sections of wild type and *Fas*-mutant mice housed at 22 °C or 4 °C for 48 h were immunostained for UCP1 (200 ×).

After prolonged exposure to 4 °C, UCP1 expression in eWAT and iWAT was significantly higher in the *Fas* mutation group compared with the control (Fig. 3).

### Relative quantification of adipose tissue-derived mRNA

**Figure 4:**
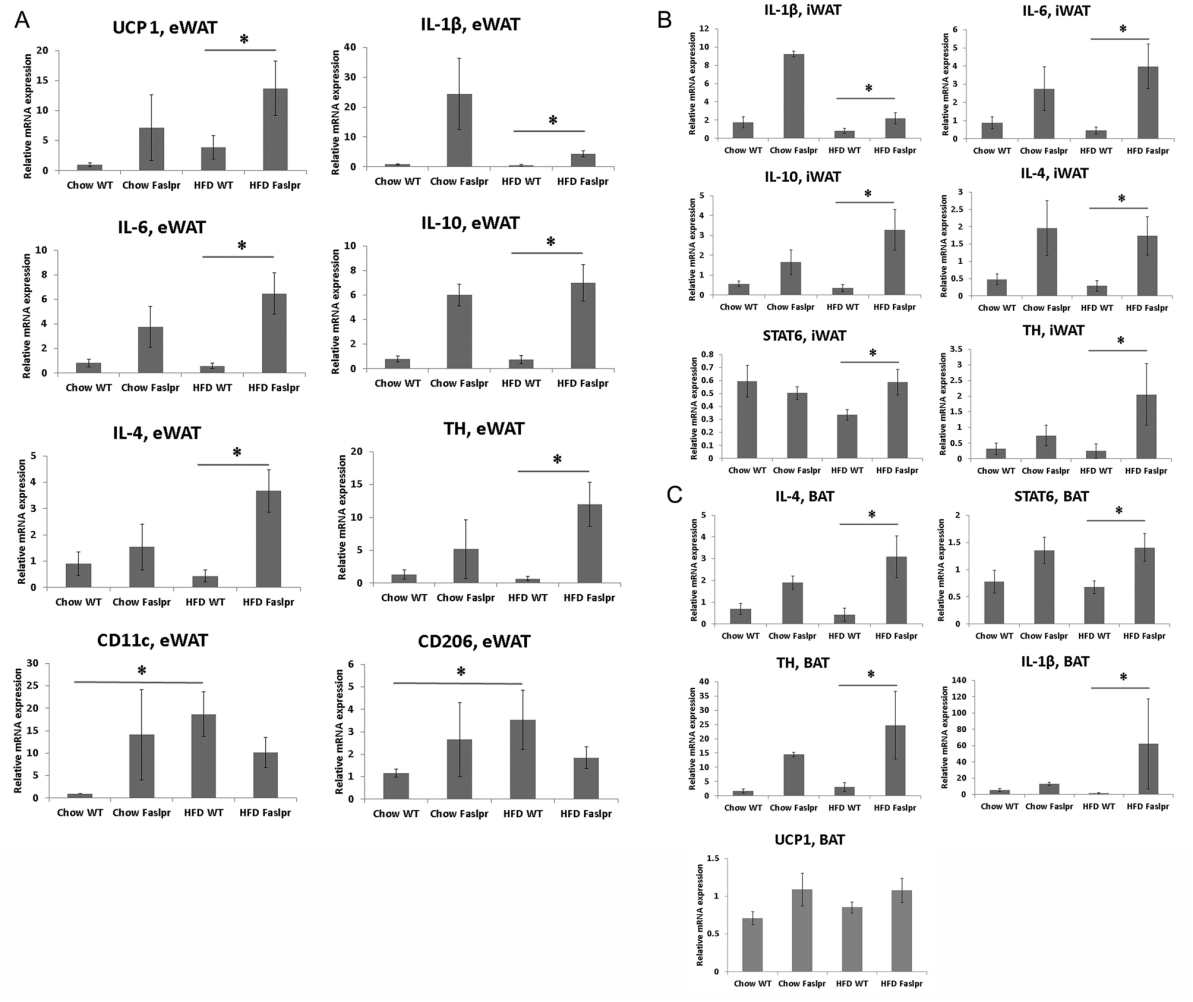
Relative quantification of mRNA using RNA extracted from adipose tissues. Relative gene expression was obtained after normalisation to GAPDH (n = 7–11 per group). The data were analysed by Mann–Whitney U test.*Significant differences (p < 0.05). *eWAT* epididymal white adipose tissue, *iWAT* inguinal white adipose tissue, *BAT* brown adipose tissue, *UCP1* uncoupling protein 1, *TH* tyrosine hydroxylase.

When mice were fed with HFD, the expression levels of UCP1, IL-1β, IL-6, IL-10, IL-4, and tyrosine hydroxylase in eWAT were significantly higher in the *Fas* mutation group than in the control group (Fig. 4A).

Although the expression levels of CD11c and CD206 in eWAT were significantly higher in WT mice fed with HFD than in those fed with chow diet, these expression levels were not significantly different between *Fas* mutation mice fed with HFD and those fed with chow diet (Fig. 4A).

When the mice were fed with chow diet, the expression of UCP1 in iWAT was significantly higher in the *Fas* mutation group than in the control group.

When mice were fed with HFD, the expression levels of IL-1β, IL-6, IL-10, IL-4, STAT-6, and tyrosine hydroxylase in iWAT were significantly higher in the *Fas* mutation group than in the control group (Fig. 4B).

When mice were fed with HFD, the expression levels of IL-4, STAT-6, tyrosine hydroxylase, and IL-1β in BAT were significantly higher in the *Fas* mutation group than in the control group (Fig. 4C). The mean UCP1 expression in BAT was higher in the *Fas* mutation group than in the control group; however, there was no statistically significant difference between the groups (Fig. 4C).

### *Fas* mutation mice were resistant to high-fat diet-induced M2 to M1 shift

Representative flow cytometry gating schemes of various cell populations in stromal vascular cells obtained from epididymal white adipose tissues are shown in the Supplementary Figs 1–4. The number of leukocytes, CD64 + F4/80 + macrophages, CD11c- CD206- macrophages, CD11c + M1 macrophages, CD206 + M2 macrophages, and dendritic cells in eWAT was significantly higher in WT and *Fas* mutation mice fed with HFD than in those fed with chow diet, respectively. The total number of CD64 + F4/80 + macrophages, CD11c− CD206− macrophages, and neutrophils in eWAT was significantly higher in HFD WT mice than in HFD *Fas* mice. When mice were fed with HFD, the number of CD11c + M1 macrophages was higher in WT mice than in *Fas* mutation mice. The ratios of M1:M2 were 0.076 ± 0.015, 0.109 ± 0.012, 1.042 ± 0.295, and 0.363 ± 0.139 in Chow WT, Chow *Fas*, HFD WT, and HFD *Fas* mice, respectively. The number of F4/80− CD137 + beige adipocytes was significantly higher in HFD *Fas* than in Chow *Fas*.

**Figure 5:**
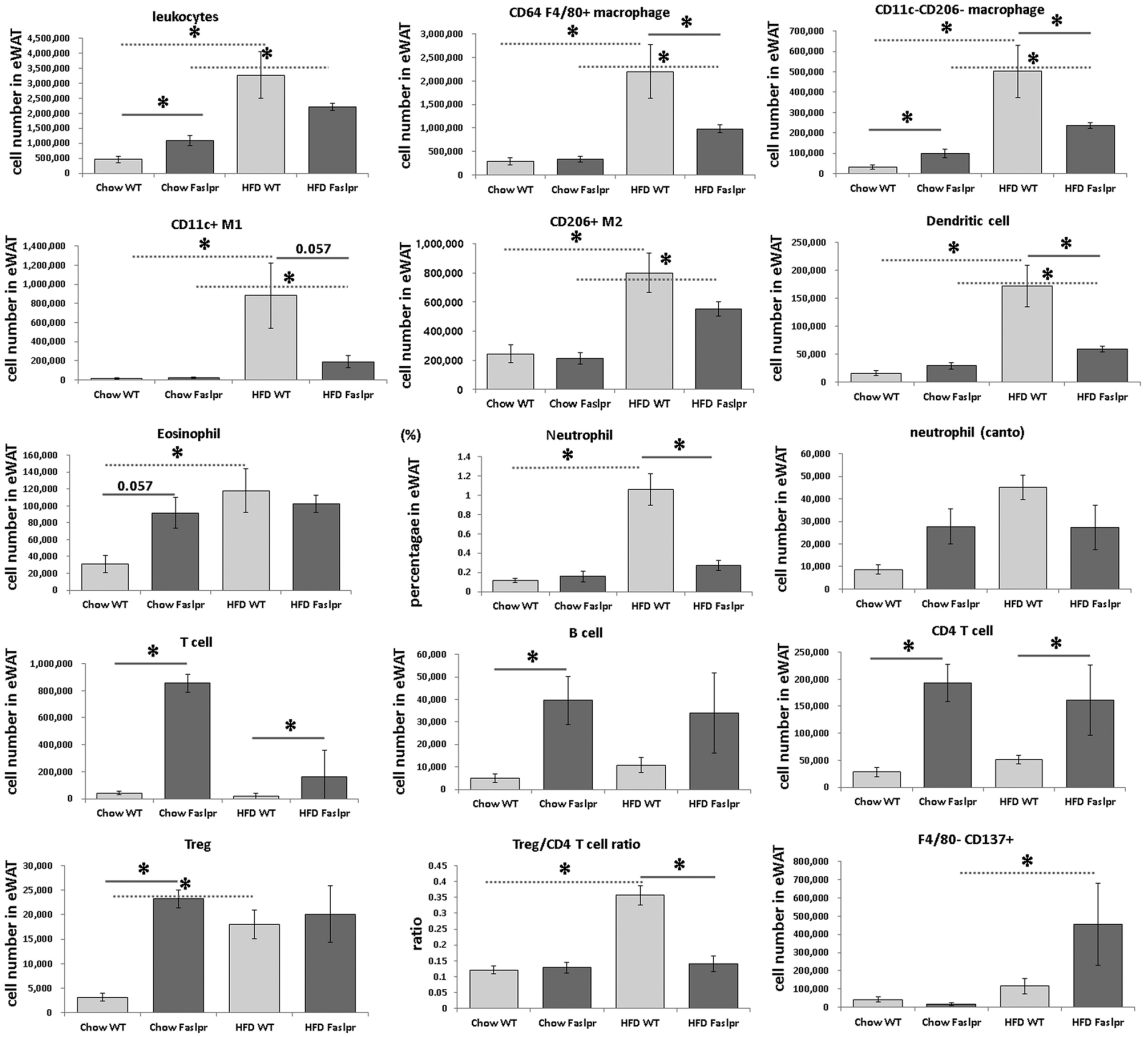
The populations of inflammatory cells in epididymal white adipose tissue analysed by flow cytometry. The numbers of leukocytes, CD64 F4/80 + macrophages, CD11c− CD206− macrophages, CD11c + M1, CD206 + M2, dendritic cells, eosinophils, neutrophils, T cells, B cells, CD4 + T cells, regulatory T cells, the Treg/CD4 + T ratio, and F4/80-CD137 + beige adipocytes per fat (epididymal white adipose tissue) (n = 4 per group). The data were analysed by the Mann–Whitney U test. *Significant differences (p < 0.05).

The numbers of eosinophils, neutrophils, and regulatory T cells (Tregs) were significantly higher in Chow *Fas* than in Chow WT, and those in HFD WT were significantly higher than in Chow WT (cells per eWAT, Fig. 5).

**Figure 6:**
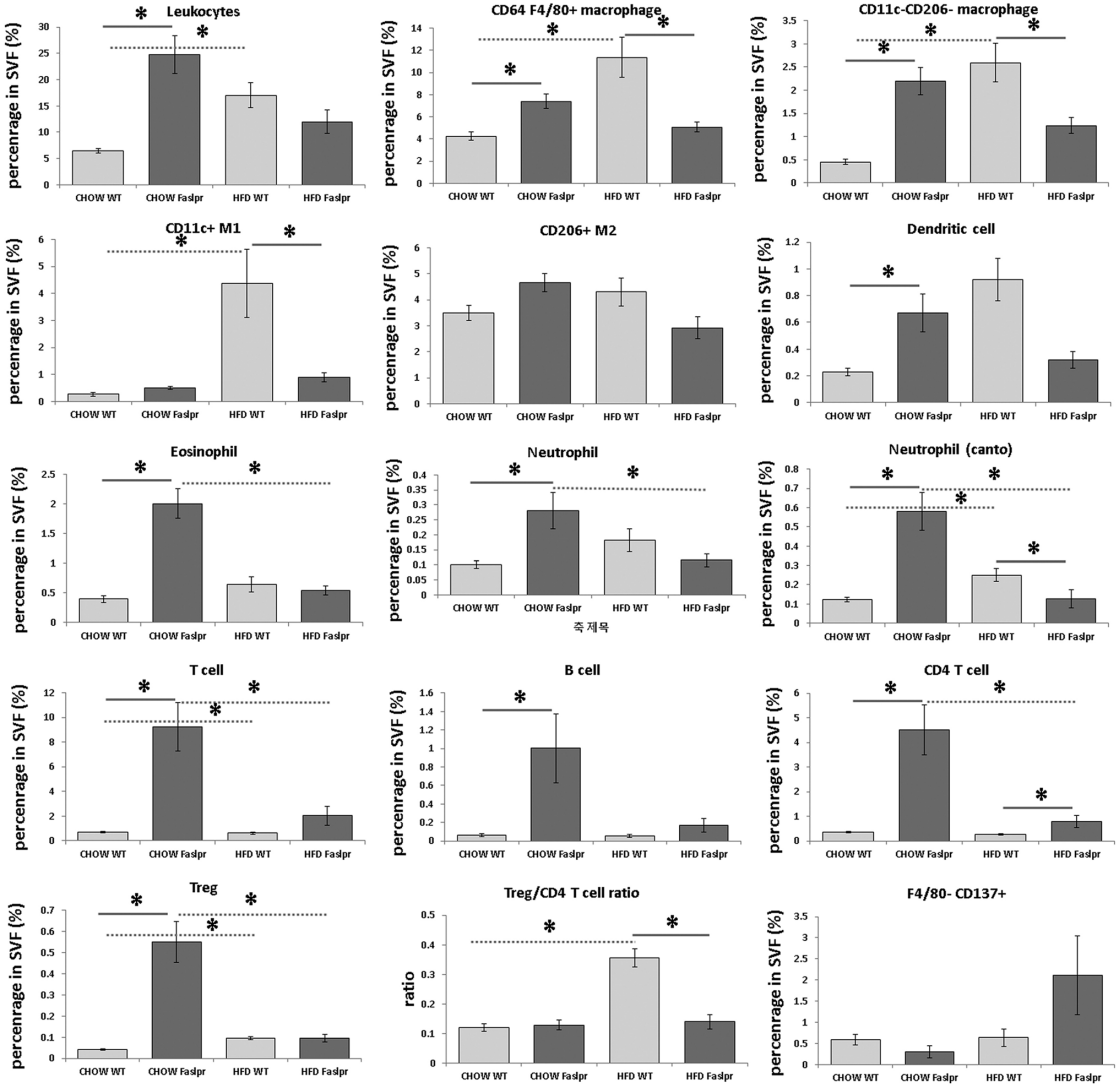
The percentages of inflammatory cells in stromal vascular cells from epididymal white adipose tissue analysed by flow cytometry. The percentages of leukocytes, CD64 + F4/80 + macrophages, CD11c− CD206− macrophages, CD11c + M1, CD206 + M2, dendritic cells, eosinophils, neutrophils, T cells, B cells, CD4 + T cells, regulatory T cells, the Treg/CD4 + T ratio, and F4/80− CD137 + beige adipocytes in stroma vascular cells from eWAT (n = 4 per group). The data were analysed by the Mann–Whitney U test. *Significant differences (p < 0.05). *eWAT* epididymal white adipose tissue, *SVF* stromal vascular cells.

**Figure 7:**
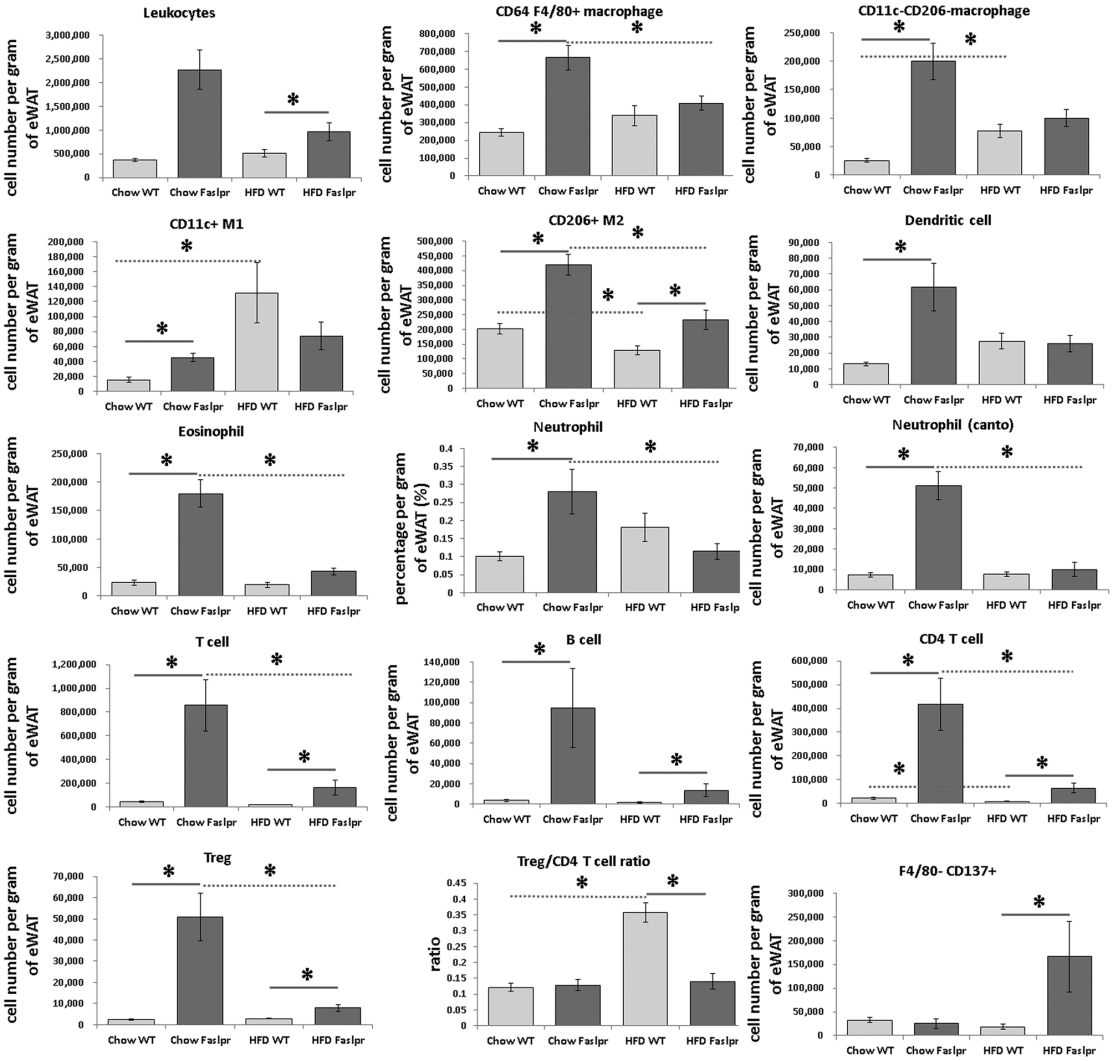
The numbers of inflammatory cells per gram of epididymal white adipose tissue analysed by flow cytometry. The numbers of leukocytes, CD64 + F4/80 + macrophages, CD11c- CD206- macrophages, CD11c + M1, CD206 + M2, dendritic cells, eosinophils, neutrophils, T cells, B cells, CD4 + T cells, regulatory T cells, the Treg/CD4 + T ratio, and F4/80- CD137 + beige adipocytes per gram of eWAT (n = 4 per group). The data were analysed by the Mann–Whitney U test. *Significant differences (p < 0.05). *eWAT* epididymal white adipose tissue.

The percentages of CD64 + F4/80 + macrophages, CD11c- CD206- macrophages, and CD11c + M1 macrophages in stroma vascular cells from eWAT were significantly higher in HFD WT mice than in HFD *Fas* mice (% in single cells, Fig. 6). The numbers of CD206 + M2 macrophages, Tregs, and eosinophils per gram of eWAT were significantly higher in Chow *Fas* than in Chow WT (cells per g, Fig. 7).

### *Fas* mutation reduced significantly the serum levels of free fatty acid, glycerol, total cholesterol, leptin, and insulin

*Fas* mutation significantly reduced the serum levels of free fatty acid and glycerol, regardless of the type of diet, when mice were housed at 22 °C (Table 1). *Fas* mutation significantly reduced the levels of total cholesterol and insulin in mice fed with HFD and housed at 22 °C (Table 1). Regardless of the type of diet (HFD or chow diet), *Fas* mutation significantly reduced the levels of leptin (Table 1). *Fas* mutation in MRL/MpJ mice significantly increased serum IL-6 and tumour necrosis factor-α (TNF-α) levels due to systemic autoimmunity and massive lymphadenopathy associated with the proliferation of aberrant T cells.

**Table 1:** Serum levels of free fatty acids, glycerol, total cholesterol, triglyceride, insulin, cytokines, and adipokines of wild type and *Fas* mutation mice.

Parameter	High fat diet		Chow diet	
	Wild type	Fas mutation	Wild type	Fas mutation
FFAs (mM)	1.1 ± 0.1	0.5 ± 0.1*	1.3 ± 0.1	0.8 ± 0.1*
Glycerol (mg/L)	59.3 ± 6.7	33.6 ± 3.7*	50.6 ± 3.3	34.4 ± 3.7*
T-Chol (mg/dl)	268.2 ± 11.5	193.0 ± 36.1*	195.1 ± 10.0	172.9 ± 36.1
TG (mg/dl)	152.9 ± 17.4	115.1 ± 12.4	241.0 ± 21.6	174.6 ± 12.4
IL-6 (pg/ml)	14.6 ± 4.7	70.4 ± 16.5*	0.1 ± 0.1	60.2 ± 15.3
TNF-α (pg/ml)	4.0 ± 1.4	53.6 ± 6.5*	2.1 ± 1.2	32.1 ± 6.0*
Insulin (pg/ml)	3,458.5 ± 477.4	1582.6 ± 479.9*	1813.8 ± 338.4	916.5 ± 444.3
Leptin (pg/ml)	16,533.5 ± 1,165.7	6,398.5 ± 655.0*	5,967.9 ± 641.2	1651.9 ± 606.5*
PAI-1 (pg/ml)	1536.8 ± 179.5	5,245.0 ± 1,640.1*	937.7 ± 41.7	2,951.1 ± 1518.4
Resistin (pg/ml)	1922.0 ± 132.3	1559.0 ± 147.3	774.9 ± 37.9	1,332.7 ± 136.3*
Adiponectin (ng/ml)	12,969.9 ± 358.0	11,200.6 ± 1,041.9	11,020.2 ± 654.9	8,396.3 ± 964.6*

Results are mean ± SEM.

*FFAs* free fatty acids, *T-chol* total cholesterol, *TG* triglyceride, *TNF-α* tumour necrosis factor-α, *PAI-1* plasminogen activator inhibitor-1.

*Significant (p < 0.05) differences from the wild type mice in same diet are marked by asterisk (Student's t-test).

## Discussion

MRL/lpr mice possessed a lean phenotype despite exhibiting severe splenomegaly, lymphadenopathy, systemic inflammatory status markers (supported by high serum TNF-α), and lymphocytic infiltration in the fat. Eosinophil and M2 macrophage levels per gram fat and the gene expressions of *IL-4* and *IL-10* in white adipose tissue were significantly higher in the *Fas*-mutant mice than in the wild type mice. Thus, these factors appear to be more involved in controlling local inflammation in fat. The results of this study using MRL/lpr mice suggested that *Fas* can be used as a therapeutic target for obesity.

Obesity is associated with high circulating leptin levels and leptin resistance in humans and rodents^20,21^. *Fas* mutation significantly reduced the levels of leptin. Triglycerides are the most important storage form of fat, which are hydrolysed to release glycerol and free fatty acids into the blood. Thus, the circulating levels of free fatty acids and glycerol are considered to reflect lipolysis. Alterations in lipolysis are typically associated with obesity. An increase in the basal rates of lipolysis might contribute to the development of insulin resistance. High-circulating levels of free fatty acids are one of the important pathogenic factors contributing to reduced insulin sensitivity and islet dysfunction in type 2 diabetes. FFA causes reduced peripheral glucose uptake through the suppression of glucose oxidation within the skeletal muscles^22^. Insulin secretion is inhibited by FFA through the uncoupling of mitochondrial metabolism in islets^23^. Increased levels of FFA induce insulin signalling impairment and insulin resistance^24^. Thus, decreased levels of FFA resulting from *Fas* mutation in HFD-fed mice appear to improve insulin sensitivity and glucose tolerance.

IL-6 exerts various effects on biological activities in its target cells and is an essential modulator of various inflammatory states. Increased serum levels of IL-6 have been observed in patients with various autoimmune disease^25^. IL-6 plays a key role in the development of murine lupus nephritis and its deficiency in MRL/lpr mice decreases lupus activity, ultimately resulting in delayed onset of lupus nephritis^26^. In a previous study, an age-associated increase in serum IL-6 levels due to abnormal transcriptional control was observed in MRL/lpr mice. Abnormal expression of IL-6 might play an important role in the activation of polyclonal B cells and the development of glomerulonephritis in MRL/lpr mice^27^.

HFD-induced obesity has been demonstrated to cause a shift in adipose tissue macrophages from the M2 polarised state in lean animals to the M1 proinflammatory state^28^; *Fas* mutation prevented HFD-induced obesity. It has been reported that insulin resistance is associated with the number of both M1 macrophages and the M1:M2 ratio^29,30^. TNF-α directly decreases insulin sensitivity and increases lipolysis in adipocytes^31^. In adipose tissue, the secreted TNF-α is primarily derived from M1 macrophages and the accumulation of M1 macrophages during obesity contributes to the development of insulin resistance^32^. In this study, a low M1:M2 ratio and enhanced glucose tolerance were both observed in *Fas*-mutant mice. When mice were fed an HFD, the gene expression of IL-4 and IL-10 in white adipose tissues were significantly higher in *Fas*-mutant mice than in WT mice. IL-4 and IL-13 induce the differentiation of M2 macrophages^29,33^, and the upregulation of IL-10 under HFD conditions is involved in M2 macrophage recruitment that contributes to reducing inflammation and improving inulin sensitivity^29,33,35^. Further, the gene expression of tyrosine hydroxylase was significantly increased in the eWAT, iWAT, and BAT of the *Fas*-mutant group compared to those in the WT group after feeding with a high-fat diet. Adipocytes appear to be able to adapt to environmental demands such as lipid overload and cold exposure by producing catecholamines^36^. Tyrosine hydroxylase is one of the essential enzymes required for the biosynthesis of catecholamines. Beige fat expressing UCP1 provides a defence against cold and obesity, and tyrosine hydroxylase is required for the development of beige fat^37^.

As cold exposure is one of the most studied stimuli that induce the browning of WAT (synthesis of beige fat), the cold challenge was performed to compare the thermogenic protein activity, UCP1 expression, and browning between the *Fas*-mutant and WT groups. In response to the cold challenge, UCP1 expression and browning were also significantly higher in *Fas*-mutant mice than in WT mice. Eosinophils, the type 2 cytokines IL-4 and IL-13, and M2 macrophages are essential for the development of functional beige fat^37^. Further, eosinophils are the major IL-4 expressing cells in white adipose tissues of mice, and they sustain M2 macrophages associated with glucose homeostasis^38^.

The numbers of CD206 + M2 macrophages, Tregs, and eosinophils per gram of eWAT were significantly higher in Chow *Fas* mice than in Chow WT mice, and this might contribute to the browning of white adipose tissues in response to the cold challenge, which is observed in *Fas*-mutant mice. As described above, the presence of eosinophils and Tregs that secrete the cytokines IL-4/IL-13 and IL-10, respectively, polarises macrophages in adipose tissues toward an anti-inflammatory phenotype (M2)^37,38^, and IL-10 upregulation contributes to reduced inflammation and an improved insulin sensitivity^34,35,39^.

Mutations are generally caused by natural events such as errors during the cloning of genetic material and by external factors such as radiation or chemical effects^40^. Mutations result in changes in the protein produced by the gene, leading to changes in the genotype. *Fas* mutation is one of the most representative spontaneous mutations. As described in the introduction section, MRL/lpr is a type of spontaneous autoimmune disease that is caused by *Fas* mutation, and mice possessing this mutation exhibit very severe autoimmune disease phenotyping compared to C3H, C57BL/6, and BALB/c background mice^41^. Additionally, fat metabolism due to *Fas* mutation could be affected by genetic background in the degree of phenotyping. In MRL/lpr background mice, serum concentrations of FFA, glycerol, leptin, and adiponectin were significantly lower and serum concentrations of resistin were significantly higher in *Fas*-mutant mice compared to those in WT mice, even with a Chow diet. Although an HFD was administered for 6 weeks, there were no significant differences in these blood parameters related to fat metabolism between AFasKO and WT C57BL/6 background mice^11^.

In summary, *Fas* mutation conveys resistance to high-fat diet-induced obesity by increasing IL-4 and IL-10 and by promoting thermogenic protein activity and browning. The results of this study also suggested that *Fas* plays a key role in obesity besides its well-known functions associated with apoptosis; our findings indicate that genetic background should be considered for research, medical prevention, and treatment. These data obtained using *Fas*-mutated mice might exhibit a translational potential for developing therapeutic strategies based on targeting of *Fas*.

## Methods

### Experimental animals

The breeding pairs of MRL/lpr and background matched control MRL/MpJ mice were purchased from Jackson Laboratory (Bar Harbor, ME, USA). This study was reviewed and approved by the Institutional Animal Care and Use Committee of Samsung Biomedical Research Institute (SBRI) in Samsung Medical Center (20140915001). SBRI is accredited by the Association for the Assessment and Accreditation of Laboratory Animal Care International and abides by the guidelines of the Institute of Laboratory Animal Resources.

### Experimental schedule

All mice (male) were housed in a specific pathogen-free environment and were fed ad libitum with a regular chow diet (protein 24.651% Kcal, Fat 13.205% Kcal, and Carbohydrate 62.144% Kcal, PicoLab^®^ Rodent Diet 20, 5,053, LabDiet, St. Louis, MO) or a high-fat diet (protein 20% Kcal, Fat 60% Kcal, and Carbohydrate 20% Kcal, rodent diet with 60 kcal % fat, D12492, Research diets, New Brunswick, NJ) from 6 weeks of age until the end of the experiments (17–19 weeks of age). Their body weight was measured once a week during the experiment period ^42^.

Food intake, activity, rearing, and energy expenditure were measured in eight mice per group by indirect calorimetry at 13–15 weeks of age.

Intraperitoneal glucose tolerance tests (IPGTT) were conducted, and body fat percentage was measured by dual-energy X-ray absorptiometry (DEXA) at 16 weeks of age. For IPGTT, mice (n = 8 per group) were subjected to fasting for 6 h, and glucose (2 g/kg body weight) was then injected intraperitoneally. The blood samples were taken at 0 min, 15 min, 30 min, 60 min, 90 min, and 120 min for blood glucose analyses using a glucometer (Accu-chek).

For cold challenge experiments, mice that were chronically housed at 22 °C were divided into two groups based on their body weights (the two groups have the same average body weight) and were housed at 22 °C or acutely shifted to 4 °C environment for 48 h. During the cold challenge experiments, mice were fed ad libitum and were housed singly in cold chambers (DBL Co. Ltd., Incheon, South Korea). At 17–18 weeks of age, adipose tissues were harvested weighed, and snap-frozen in liquid nitrogen for RNA expression analysis, or they were fixed in 10% neutral buffered formalin for histological examination. Relative sizes of adipocytes were measured using Image J (Version 1.45 s, NIH, USA) according to the method described in a previous study^43^. After H&E staining, images were taken under a microscope (200 ×) and stored on a computer. The sizes of the adipocytes were analysed using the ImageJ version 1.53a program (https://imagej.nih.gov/ij) with the Adiposoft version 1.16 plug-in (https://imagej.net/adiposoft). The following is a detailed description of the procedure for setting up and running the ImageJ program: (1) Go to the Plugins menu in ImageJ, and click on Adiposoft (Plugins → Jars → Adiposoft). (2) Choose the Auto mode and the Exclude on edges parameters. (3) Set the parameters of minimum diameter as (30) and maximum diameter as (3,000), considering the wide size range of the adipocytes in this study. (4) Select the directory that contains the images to analyse. (5) Choose the output directory where the results of the analysis to be stored. (6) The program analyses the images in completely automated mode and stores the results in the output directory; adipocytes were counted and the absolute pixel area of each object was calculated. The area was calculated only in the part that completely constituted one cell and the average area was acquired by the results.

### Body temperature measurement

Before autopsy, the body temperature was measured using a monitoring system (Model 1025T, Small Animal Instruments, Inc., Stony Brook, NY, USA); body temperature was measured using a small rectal temperature probe. The probe was inserted into the rectum, and the body temperature was recorded when the temperature stopped fluctuating.

### Relative quantification of mRNA using RNA extracted from adipose tissues

Total RNA from fat tissues was extracted using an RNeasy Lipid Tissue Mini Kit (Qiagen, Valencia, CA, USA). RNA was reverse transcribed using the amfiRivert cDNA Synthesis Master Mix (GenDEPOT, Barker, TX, USA). The following primers were used to amplify cDNA fragments according to previous studies: Gapdh: forward 5′-TGCACCACCAACTGCTTAGC-3′ and reverse 5′-TCTTCTGGGTGGCAGTGATG-3′; UCP-1: forward 5′-GATGGTGAACCCGACAACTT-3′ and reverse 5′-CTGAAACTCCGGCTGAGAAG-3′; IL-1β: forward 5′-CTGGTGTGTGACGTTCCCATTA-3′ and reverse 5′-CCGACAGCACGAGGCTTT-3′; IL-6: forward 5′-CCAGTTGCCTTCTTGGGACT-3′ and reverse 5′-GGTCTGTTGGGAGTGGTATCC-3′; IL-10: forward 5′-GCTCTTACTGACTGGCATGAG-3′ and reverse 5′-CGCAGCTCTAGGAGCATGTG-3′; IL-4: forward 5′-GGTCTCAACCCCCAGCTAGT-3′ and reverse 5′-GCCGATGATCTCTCTCAAGTGAT-3′; STAT6: forward 5′-CTGGGGTGGTTTCCTCTTG-3′ and reverse 5′-TGCCCGGTCTCACCTAACTA-3′; Tyrosine hydroxylase: forward 5′-CCCCACCTGGAGTACTTTGTG-3′ and reverse 5′-CTTGTCCTCTCTGGCACTGC-3′; CD11c: forward 5′-CTTGTCCTCTCTGGCACTGC-3′ and reverse 5′-GCACACTGTGTCCGAACTC-3′; CD206: forward 5′-CAGGTGTGGGCTCAGGTAGT-3′ and reverse 5′-CAGGTGTGGGCTCAGGTAGT-3′. Relative gene expression was obtained after normalisation to GAPDH. During the triplicate analysis, the Ct value of GAPDH was obtained very stably in each sample. Mean Ct values of GAPDH in eWAT from CC, CF, HC, and HF groups were 25.3 ± 1.73, 27.5 ± 1.8, 25.8 ± 2.2, and 28.5 ± 2.6, respectively. Mean Ct values of GAPDH in iWAT from CC, CF, HC, and HF groups were 24.1 ± 0.4, 23.9 ± 0.6, 22.0 ± 0.4, and 24.9 ± 0.7, respectively. Mean Ct values of GAPDH in BAT from CC, CF, HC, and HF groups were 24.1 ± 0.4, 25.2 ± 0.4, 23.2 ± 0.5, and 24.7 ± 0.2, respectively.

### Flow cytometry

The populations of inflammatory cells in eWAT from four mice per group were analysed by flow cytometry at 19 weeks of age.

To stain the immune cells in mouse adipose tissue, antibodies specific for CD16/32 (clone; 93, cat number cat. 101320), CD11b (clone; M1/70, cat. 101216 and 101226), CD11c (clone; N418, cat.117308, 117318), CD19 (clone; 6D5, cat. 115529), CD206 (clone; C068C2, cat. 141710), CD3 (clone; 17A2, cat. 100203), CD45 (clone: 30-F11, cat. 103130), CD64 (clone; X54-5/7.1, cat.139305), F4/80 (clone; BM8, cat. 123113), I-A/I-E (clone; M5/114.15.2, cat. 107628), Ly6G (clone; 1A8, cat. 127613), and NK1.1 (clone; PK136, cat. 108708) were obtained from BioLegend (San Diego, CA, USA). CD137 (clone; 17B5, cat. 17-1371-80) and Foxp3 (clone FJK-16s, cat. 17-5773-82) staining antibodies were purchased from eBioscience (San Diego, CA, USA). Antibodies against CD4 (clone; GK1.5, cat. 553730) and Siglec-F (clone; E50-2440, cat. 562681) were obtained from BD Pharmingen (San Jose, CA, USA). The anti-F4/80 antibody (clone; A3-1, cat. MCA497PB) was obtained from Serotec (Oxford, UK). Collagenase II was obtained from Sigma-Aldrich (cat. C6885, St Louis, MO, USA) and was diluted to 400 U/mL using phosphate-buffered saline (PBS) containing Ca^2+^ and Mg^2+^ prior to use. The Foxp3 transcription factor-staining buffer set was purchased from eBioscience (cat. 00-5523-00).

To obtain the stromal vascular fraction (SVF) from mouse epididymal adipose tissue, the white fat was isolated from the adipose tissue after being placed in 10 mL fresh cold PBS. The adipose tissue was gently chopped into 1–2 mm pieces and then mixed with collagenase II solution (400 U/mL). The mixture was incubated at 37 °C for 16 min with gentle shaking. After isolation, SVF was separated from the adipocytes using centrifugation at 300 rcf for 7 min. Fc blocking antibody was used to prevent non-specific binding. Next, the SVF cells were incubated with antibodies for surface staining at 4 °C for 30 min. The cells were washed in PBS with 2% foetal bovine serum. For staining of the Foxp3 transcription factor, the cells were fixed and permeabilised with the Foxp3 transcription factor-staining buffer set and then stained with Foxp3 antibody. The dilution of all antibodies was 1:400. A LSR II (BD Biosciences) instrument and FlowJo software (Tree Star Inc, Ashland, OR, USA) were used for flow cytometry analysis (CD45 + CD64 + : macrophage, CD45 + CD64 + CD11c + CD206−: M1, CD45 + CD64 + CD11c− CD206 + : M2, CD45 + CD64− CD11c + MHC II + : dendritic cells, CD45 + CD11c− Siglec-F + : eosinophil, CD45 + CD11c− Siglec-F− Ly6G + : neutrophil, CD45 + CD11c− Siglec-F− Ly6G− NK1.1 + CD3−: NK cell, CD45 + CD11c− Siglec-F− Ly6G− NK1.1 + CD3 + : NKT cell, CD45 + CD11c− CD11b− CD3 + CD19− : T cell, CD45 + CD11c− CD11b− CD3− CD19 + : B cell, CD45 + CD11c− CD11b− CD3 + CD4 + : CD4 + T cell, CD45 + CD11c− CD11b− CD3 + CD4 + Foxp3 + : Treg, F4/80− CD137 + : beige adipocytes).

### Immunohistochemistry

For UCP1 immunohistochemistry, sections of adipose tissues were incubated with anti-UCP1 in the manner described below. Specifically, tissues were fixed in 10% neutral buffered formalin (NBF), embedded in paraffin, and sliced into 4 µm sections. Tissue sections were then deparaffinised in xylene, rehydrated in graded alcohol, and transferred to 0.01 M phosphate-buffered saline (PBS, pH 7.4). To reveal hidden antigen epitopes, heat-induced epitope retrieval (HIER) was performed by boiling the sections in EDTA buffer (pH 9.0; Dako, Carpinteria, CA) for 3 min at 121 °C. After washing in PBS buffer, endogenous peroxidase was blocked with 3% hydrogen peroxide in PBS for 10 min at room temperature. After washing in PBS buffer, the sections were treated with serum-free blocking solution (Dako) for 20 min at room temperature to block nonspecific binding. Subsequently, the sections were incubated with anti-UCP1 rabbit polyclonal antibody (ab10983, 1:1,000; Abcam, Cambridge, MA) overnight at 4 °C. After washing in PBS, the sections were incubated for 30 min at room temperature with HRP-labelled polymer conjugated secondary antibodies against rabbit IgG (Dako). Colour reactions were developed using the ready-to-use 3,3′-diaminobenzidine (DAB) substrate-chromogen solution (Dako) for 3 min, and the sections were washed with distilled water. Finally, the sections were lightly counterstained with Mayer’s haematoxylin (Dako) for 30 s before dehydration and mounting. Further, all sections were stained with H&E (BBC Biochemical, Mount Vernon, WA, USA) for histological evaluation.

### Determination of serum levels of total cholesterol, triglyceride, free fatty acid, and glycerol

Serum levels of total cholesterol and triglyceride were measured using a DRI-CHEM 3000 Colorimetric Analyzer (Fujifilm, Tokyo, Japan). Serum levels of free fatty acid and glycerol were measured using a Free Fatty Acid Quantification Kit (ab65341, Abcam) and a Glycerol Colorimetric Assay Kit (10010755, Cayman chemical., Ann Arbor, MI, USA), respectively.

### Determination of serum levels of adipokines

Serum samples from all mice were assayed using multiplex adipokine kits for insulin, leptin, resistin, IL-6, TNF-α, MCP-1, plasminogen activator inhibitor-1 (PAI-1), and adiponectin (MADCYMAG-71K and MADPNMAG-70K-01, Millipore, Bedford, MA, USA).

### Statistical analysis

All results are expressed as the mean ± standard error of the mean (SEM). The results from the experiments using quantitative RT-PCR and flow cytometry were compared between two groups using the Mann–Whitney U test. The data from calorimetry assays were analysed using repeated measured one-way analysis of variance (ANOVA). Other data were analysed using student t-tests. Differences possessing a confidence level of 95% or higher were considered statistically significant (*p* < 0.05). *Significant (*p* < 0.05) differences from the WT mice are marked by an asterisk. All statistical analyses were conducted using SPSS version 21.0 (SPSS Inc., Chicago, IL, USA).

## Supplementary information

Supplementary information
