# Supplementary material for: Fas mutation reduces obesity by increasing IL-4 and IL-10 expression and promoting white adipose tissue browning
Source: Sci Rep. 2020 Jul 20;10:12001. doi: 10.1038/s41598-020-68971-7 (PMC7371740; doi:10.1038/s41598-020-68971-7)
Supplement: Supplementary file 1 — Supplementary information [file 41598_2020_68971_MOESM1_ESM.pdf]

Supplementary information

Title:

Fas mutation reduces obesity by increasing IL-4 and IL-10 expression and promoting white adipose tissue browning

Authors:

Eun Wha Choi[1,2,*], Minjae Lee[2], Ji Woo Song[2], Kyeongdae Kim[3], Jungmin Lee[2,4], Jehoon Yang[2], Seo Hyun Lee[4], Il Yong Kim[4], Jae-Hoon Choi[3], Je Kyung Seong[4,5,*]

[1]Department of Veterinary Clinical Pathology, College of Veterinary Medicine & Institute of Veterinary Science, Kangwon National University, 1 Kangwondaehak-gil, Chuncheon, Gangwon-do, 24341, Republic of Korea

[2]Laboratory Animal Research Center, Samsung Biomedical Research Institute, Samsung Medical Center, 81 Irwon-ro, Gangnam-gu, Seoul, 06351, Republic of Korea

[3]Department of Life Science, College of Natural Sciences, Hanyang University, 222 Wangsimni-ro, Seongdong-gu, Seoul, 04763, Republic of Korea

[4]Laboratory of Developmental Biology and Genomics, BK21 Plus Program for Advanced Veterinary Science, Research Institute for Veterinary Science, College of Veterinary Medicine, and Korea Mouse Phenotyping Center , Seoul National University, 1 Gwanak-ro, Gwanak-gu, Seoul, 08826, Republic of Korea

[5]Interdiscplinary Program for Bioinformatics, Seoul National University, 1 Gwanak-ro, Gwanak-gu, Seoul, 08826, Republic of Korea

*Corresponding author: Eun Wha Choi, DVM, PhD, Assistant Professor

Address: Department of Veterinary Clinical Pathology, College of Veterinary Medicine & Institute of Veterinary Science, Kangwon National University, 1 Kangwondaehak-gil, Chuncheon-si, Gangwon-do 24341, Republic of Korea

Telephone: 82-33-250-8794

Fax: 82-33-259-5625

E-mail: ewchoi@kangwon.ac.kr

*Corresponding author: Je Kyung Seong, DVM, PhD, Professor

Laboratory of Developmental Biology and Genomics, BK21 Program for Veterinary Science, Institute for Veterinary Science, College of Veterinary Medicine, Seoul National University, 599 Gwanak-ro, Gwanak-gu, Seoul, 08826, Republic of Korea

Korea Mouse Phenotyping Center (KMPC), Seoul National University, 1 Gwanak-ro, Gwanak-gu, Seoul, 08826, Republic of Korea.

Telephone: 82-2-880-1259

Fax: 82-2-875-8395

E-mail: snumouse@snu.ac.kr

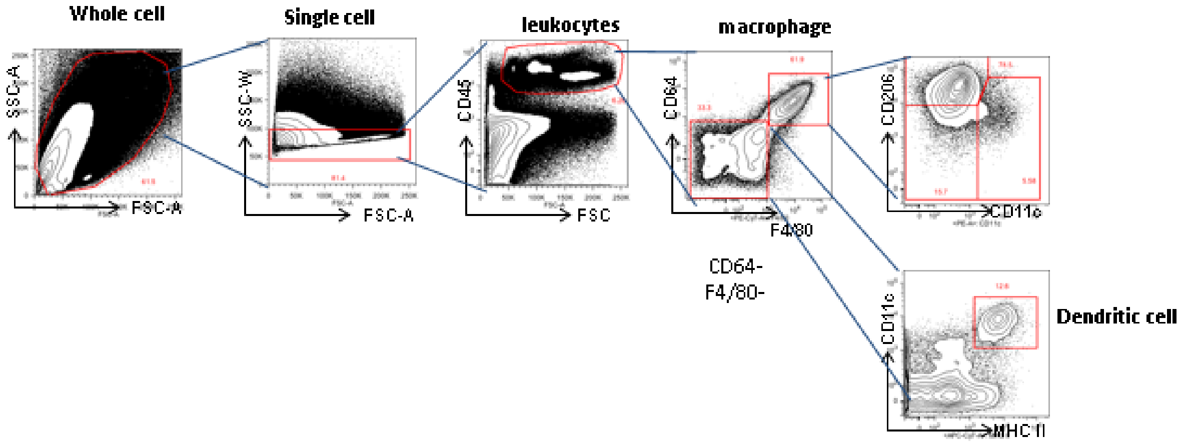

Supplementary Figure 1. A representative gating scheme of dendritic cell and macrophage populations in the epididymal white adipose tissues. For analysis of dendritic cell and macrophage populations, stromal vascular cells obtained from epididymal white adipose tissues were stained with FITC–conjugated anti-CD206, PE–conjugated anti-CD11c, PerCP–conjugated anti-CD45, PE-Cy7–conjugated anti-F4/80, APC–conjugated anti-CD64 and APC-Cy7–conjugated anti-MHC class II antibodies.

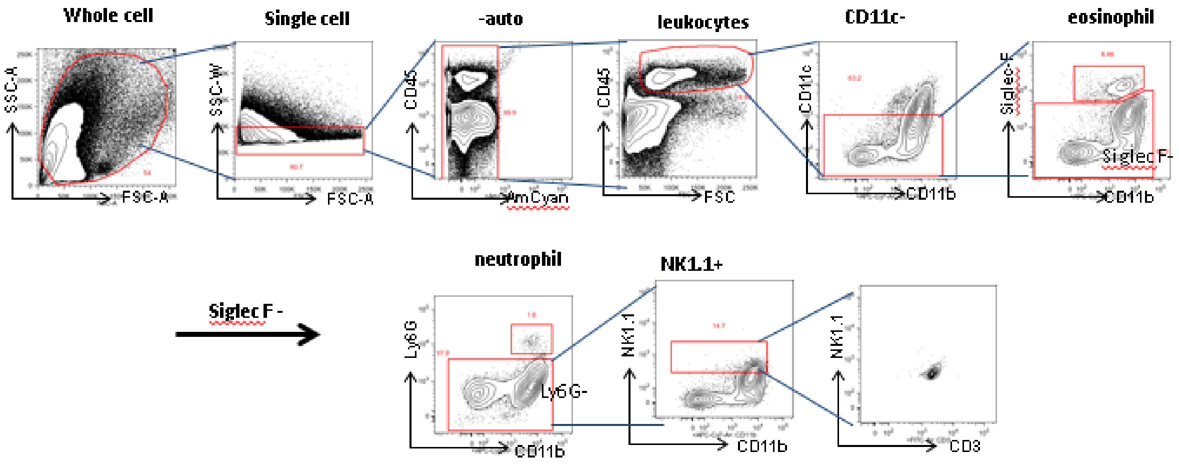

Supplementary Figure 2. A representative gating scheme of eosinophil, neutrophil, NK cell and NKT cell populations in the epididymal white adipose tissues. For analysis of eosinophil, neutrophil, NK cell and NKT cell populations, stromal vascular cells obtained from epididymal white adipose tissues were stained with FITC–conjugated anti-CD3, PE–conjugated anti-NK1.1, PerCP–conjugated anti-CD45, PE-Cy7–conjugated anti-CD11c, APC–conjugated anti-Ly6G and APC-Cy7–conjugated anti-CD11b and BV421–conjugated anti-Siglec-F antibodies.

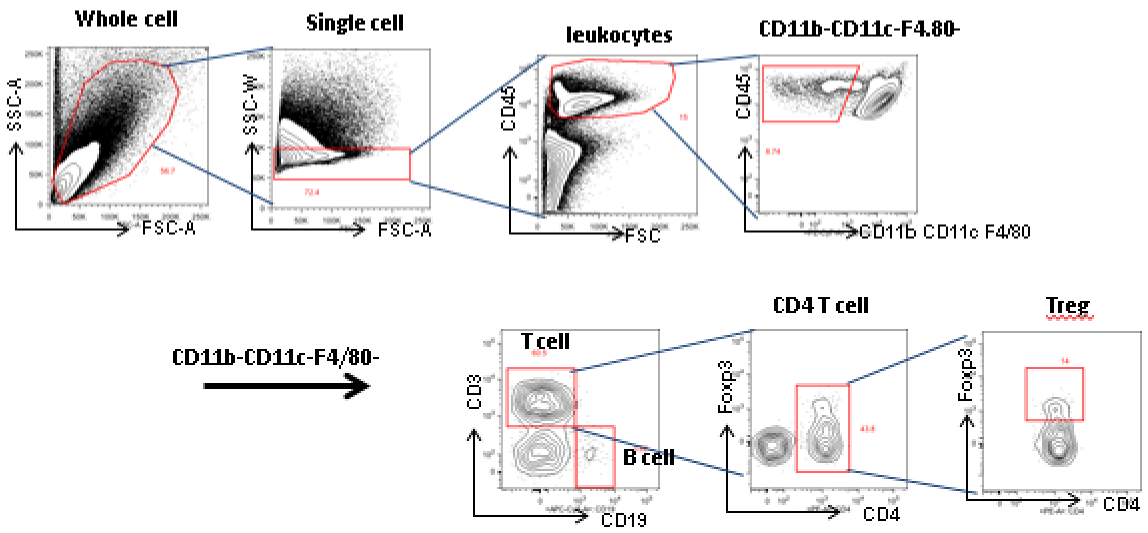

Supplementary Figure 3. A representative gating scheme of T cell, B cell, Treg cell populations in the epididymal white adipose tissues. For analysis of T cell, B cell, Treg cell populations, stromal vascular cells obtained from epididymal white adipose tissues were stained with FITC–conjugated anti-CD3, PE–conjugated anti-CD4, PerCP–conjugated anti-CD45, PE-Cy7–conjugated anti-CD11b, CD11c, F4/80, APC–conjugated anti-Foxp3 and APC-Cy7–conjugated anti-CD19 antibodies.

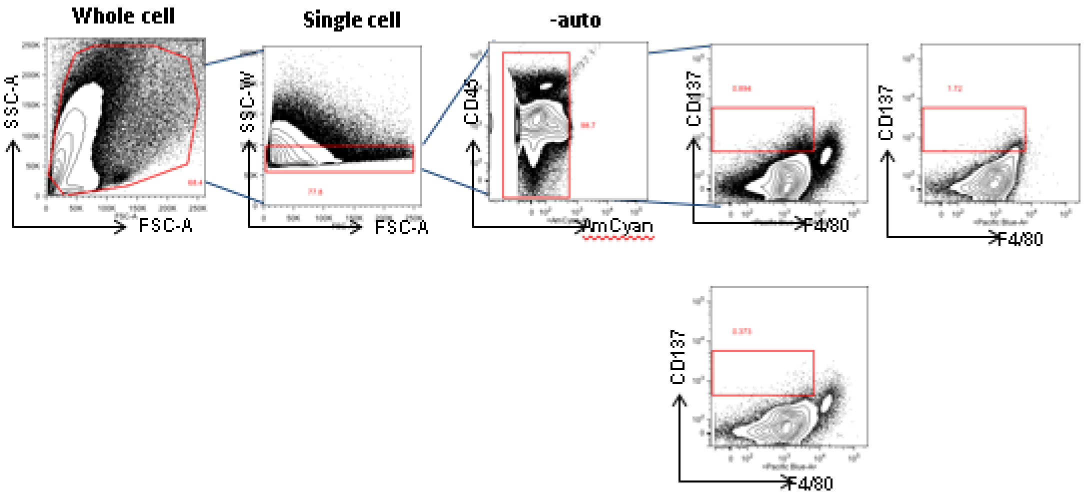

Supplementary Figure 4. A representative gating scheme of F4/80- CD137+ cell populations in the epididymal white adipose tissues. For analysis of F4/80- CD137+ cell populations, stromal vascular cells obtained from epididymal white adipose tissues were stained with APC–conjugated anti-CD137 and Pacific blue–conjugated anti-F4/80 antibodies.
